# Wound Healing Effects of* Prunus yedoensis* Matsumura Bark in Scalded Rats

**DOI:** 10.1155/2017/7812598

**Published:** 2017-03-19

**Authors:** Jin-Ho Lee, Kyungjin Lee, Mi-Hwa Lee, Bumjung Kim, Khanita Suman Chinannai, Heseung Hur, Inhye Ham, Ho-Young Choi

**Affiliations:** ^1^Department of Science in Korean Medicine, Graduate School, Kyung Hee University, Seoul 02447, Republic of Korea; ^2^Department of Herbology, College of Korean Medicine, Kyung Hee University, Seoul 02447, Republic of Korea

## Abstract

Pruni Cortex has been used to treat asthma, measles, cough, urticaria, pruritus, and dermatitis in traditional Korean medicine. The objective of this study was to investigate the effects of* Prunus yedoensis* Matsumura bark methanol extract (PYE) on scald-induced dorsal skin wounds in rats. Scalds were produced in Sprague-Dawley rats with 100°C water and treated with 5% and 20% PYE (using Vaseline as a base), silver sulfadiazine (SSD), and Vaseline once a day for 21 days, beginning 24 hours after scald by treatment group allocation. The PYE-treated groups showed accelerated healing from 12 days after scald, demonstrated by rapid eschar exfoliation compared to the control and SSD groups. PYE-treated groups showed higher wound contraction rates and better tissue regeneration in comparison with the control group. Serum analysis showed that transforming growth factor beta 1 and vascular endothelial growth factor levels remained high or gradually increased up to day 14 in both PYE groups and then showed a sharp decline by day 21, implying successful completion of the inflammatory phase and initiation of tissue regeneration. These findings suggested that PYE is effective in promoting scald wound healing in the inflammation and tissue proliferation stages.

## 1. Introduction

Burn injuries cause considerable morbidity, mortality, and socioeconomic losses. Scalds, burns caused by hot liquids, are one of the most frequently reported forms of burn. In particular, more than 50% of preschool pediatric burn patients are scald injury patients [1]. Burn injuries, including scalds, result in mild-to-severe scars on the skin. Thus, patients continuously experience serious psychological distress as well as physical pain. Therefore, better acute care and therapeutics are needed in burn management.

Many researchers around the world are investigating the pathophysiology of burns and determining the effects of new and old treatments [2]. Traditional treatment using herbal medicine could also be an effective therapy for burns.* Aloe vera* and potato are typical herbal medicines used to treat thermal burns [3, 4].

*Prunus yedoensis* Matsumura (PY) is one of the useful medicinal plants native to Jeju Island in Korea. The bark of this plant has been used to treat asthma, measles, cough, urticaria, pruritus, and dermatitis in traditional Korean medicine [5]. Recently, it was discovered to have a vasorelaxant effect. This effect is exerted via activation of the nitric oxide- (NO-) cGMP pathway and NO formation from L-arginine, which blocks the entry of extracellular Ca^2+^ into cells via receptor-operated and voltage-dependent Ca^2+^ channels, as demonstrated in rat isolated thoracic aorta [5]. In addition, the bark has an anti-inflammatory effect via the inhibition of nuclear factor-kappa B in lipopolysaccharide- (LPS-) induced RAW 264.7 macrophage cells [6] and the inhibition of LPS-induced inflammatory cytokine synthesis via I*κ*B*α* degradation and MAPK activation in macrophages [7].

As described above, the bark of PY (BPY) has been used to treat inflammatory skin diseases in traditional medicine, and recent studies have demonstrated the anti-inflammatory effects of BPY. Thus, we hypothesized that BPY might be useful to treat skin damage caused by scalds.

Therefore, the purpose of this study was to determine the effects of* P. yedoensis* Matsumura bark methanol extract (PYE) on scald wound healing through investigating the histopathological characteristics of the wounded area and serum levels of inflammatory cytokines and angiogenesis factors.

## 2. Materials and Methods

### 2.1. Preparation of PYE

Dried BPY was purchased from a herbal drug company (DongWooDang Pharmacy Co., Ltd., Korea). A voucher specimen (number PY 002) of BPY was deposited at the laboratory of herbology, College of Korean Medicine, Kyung Hee University, Seoul, Korea.

A crude extract was prepared by a decoction of dried BPY (3 kg) in 100% methanol three times (3 h per time) using a heating mantle-reflux. After filtering with filter paper, the extract was evaporated using a rotary vacuum evaporator (N-N series, Eyela Co., Ltd., Japan) and lyophilized using a freeze-dryer (OPR-FD4-8612 model, Operon Co., Ltd., Korea). The yield of crude extract was 27%.

### 2.2. Animals

Male Sprague-Dawley (SD) rats (6 weeks old, weight 180–220 g; Samtaco Co., Ltd., Korea) were housed under controlled conditions (22 ± 2°C; lighting, 07:00–19:00) with food and water available ad libitum.

### 2.3. Induction and Treatment of Scald Wounds

All experiments were performed according to the animal welfare guidelines issued by the Kyung Hee University Institutional Animal Care and Use Committee (protocol approval number KHUASP (SE)-13-003). Scald wounds were induced on the backs of the SD rats using previously described methods [8]. After scalding, rats were randomly divided into 5 groups (control, silver sulfadiazine, Vaseline, 5% PYE, and 20% PYE, *n* = 8, resp.) and, after 24 hours, 0.5 g of the experimental substances was applied to the scalded area once a day for 3 weeks. The control (CON) group was not treated after scald. The silver sulfadiazine (SSD) group was treated with the reference standard of a 1% (w/w) SSD cream, the Vaseline group was treated with Vaseline, and the 5% and 20% PYE groups were treated with Vaseline-based 5% and 20% (w/w) PYE ointments, respectively.

### 2.4. Evaluation of Wound Healing Potential

The wound area was photographed every 3 days and analyzed using ImageJ (Broken Symmetry Software). The percentage of the wound contracture rate was calculated using the following formula: % contracture = specific day wound size/initial wound size × 100.

IL-10, transforming growth factor-*β*1 (TGF-*β*1), and vascular endothelial growth factor (VEGF) levels in rat serum were measured using the same methods as in our previous study [8].

For histopathological examinations, rats were euthanized at days 2, 14, and 21 (*n* = 1, 1, and 4, resp.) after scald using ether, and skin samples were taken. After fixation using a 10% formalin solution, the tissue was washed in running tap water, dehydrated in ascending grades of ethyl alcohol, and cleared in xylene. The tissue was placed in paraffin, cut into 6 *μ*m thick slices using a microtome and stained with hematoxylin and eosin (H&E). Masson-Goldner trichrome was utilized for histological studies, including evaluation of the extent of reepithelialization, maturation, and organization of the epidermis, granulation tissue formation, collagenization, and inflammatory cells and scar formation in the dermis.

In the current study, the same CON, SSD, and Vaseline treated data as the data described in the previous study [8] were used.

### 2.5. Statistical Analysis

Data were expressed as the mean ± standard error of the mean. Statistical comparisons were made using one-way analysis of variance followed by Tukey's post hoc test using SPSS (version 13.0) statistical analysis software (IBM Inc., IL, USA).* P* values less than 0.05 were considered to be statistically significant.

## 3. Results and Discussion

### 3.1. Clinical Assessment

**Figure 1:**
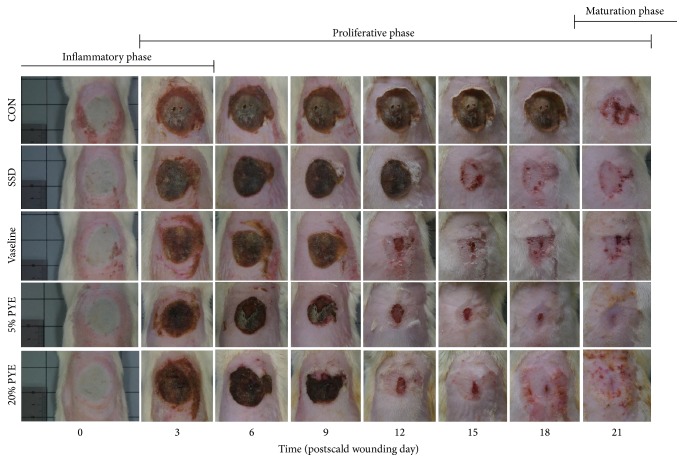
Gross appearance of the scald wounds on days 0, 3, 6, 9, 12, 15, 18, and 21. CON, control; SSD, silver sulfadiazine; PYE,* Prunus yedoensis *Matsumura bark methanol extract.

**Figure 2:**
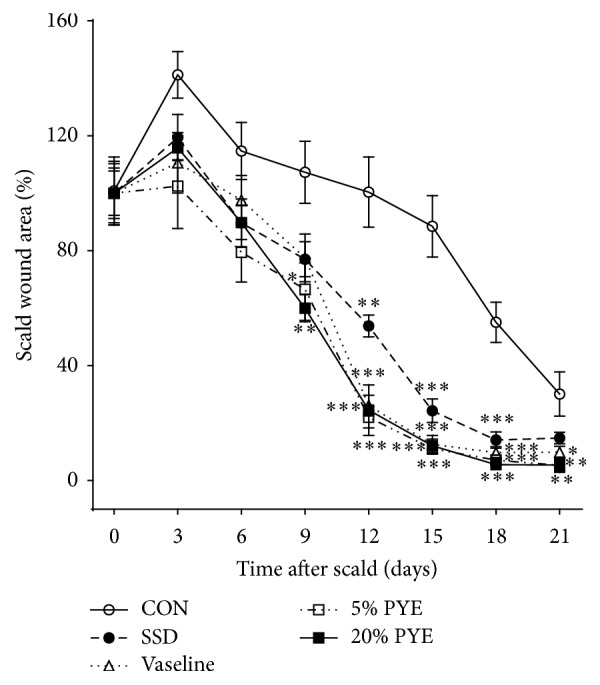
Changes in scald wound sizes. The percentage of wound contracture rate was calculated using the following formula: % contracture = specific day wound size/initial wound size × 100. CON, control; SSD, silver sulfadiazine; PYE,* Prunus yedoensis *Matsumura bark methanol extract. Values were expressed as the mean ± standard error of the mean (*n* = 6). ^*∗*^*P* < 0.05, ^*∗∗*^*P* < 0.01, and ^*∗∗∗*^*P* < 0.001 versus control.

From day 3 to day 9, all groups exhibited thick scabs with red rims along the margins. On day 12, the scabs were falling off from the wound surface and wound sizes were decreased in Vaseline and PYE groups but thick and dark brown scabs still remained in SSD and control groups. On days 18 and 21, the wound surfaces were almost closed and wound sizes were smallest in PYE groups. During the experiment periods, all groups that had received some form of ointment treatment showed significantly faster-wound healing process than the control group. In particular, PYE groups showed the fastest wound healing processes (Figures 1 and 2).

### 3.2. Histopathological Results

**Figure 3:**
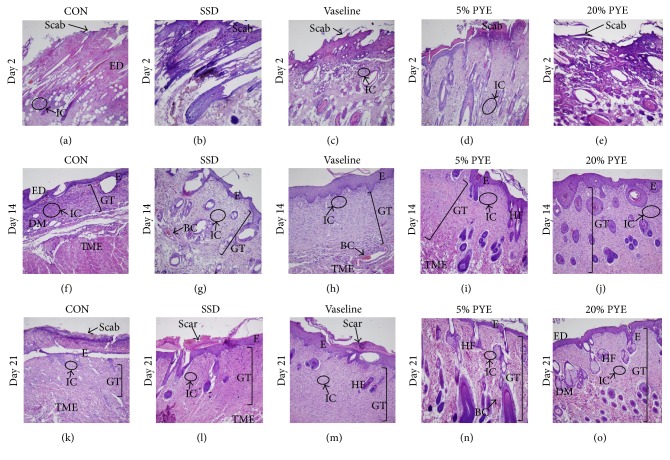
Histological appearance of scald wounds stained with hematoxylin and eosin on days 2, 14, and 21. Magnification: ×100. CON, control; SSD, silver sulfadiazine; PYE,* Prunus yedoensis *Matsumura bark methanol extract; ED, epidermis; IC, inflammatory cells; GT, granulation tissue; DM, dermis; E, epithelialization; TME, tunica muscularis externa; HF, hair follicle; BC, blood capillaries.

**Figure 4:**
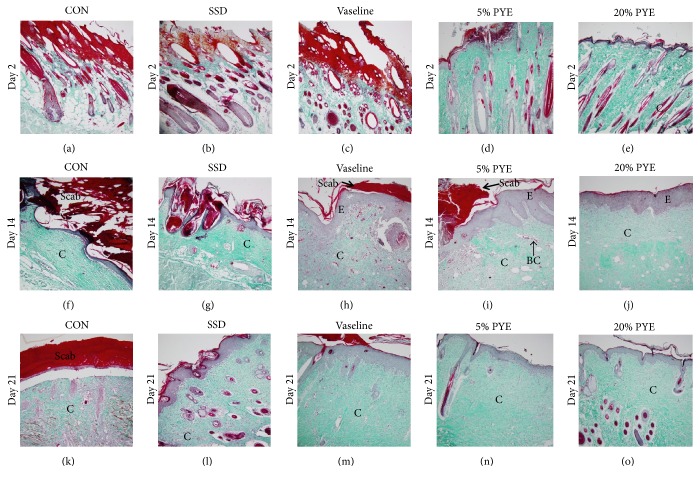
Histological appearance of scald wounds stained with Masson-Goldner trichrome stain on days 2, 14, and 21. Magnification: ×100. CON, control; SSD, silver sulfadiazine; PYE,* Prunus yedoensis *Matsumura bark methanol extract; C, collagen; E, epithelialization; BC, blood capillaries.

No epidermal regeneration was observed on day 2 (Figures 3(a)–3(e) and Figures 4(a)–4(e)). On day 14, the Vaseline group and PYE groups showed significantly better epithelialization than the control group (Figures 3(h)–3(j); Figures 4(h)–4(j)).

On day 2, all rats exhibited damage to the epidermis, dermis, and subcutaneous tissue. On day 14, a remarkably significant increase in the quantity of granulation tissue was observed in both PYE groups compared to the control group and the Vaseline group (Figures 3(f)–3(j)). The 20% PYE group showed the thickest granulation tissue (Figure 3(j)). In contrast, the granulation tissue observed in the control group was uneven and patchy (Figure 3(f)). And significant masses of granulation tissue were observed in the PYE-treated groups on day 21 (Figures 3(n) and 3(o)). Granulation tissue can be found in secondary intention healing and is dense with blood vessels, macrophages, and fibroblasts embedded in a loose matrix of collagen and fibronectin [9]. Thus, granulation tissue is one of the important factors in the wound healing processes. These results suggest that the wound healing effect of PYE application is related to the activation of granulation tissue formation.

On day 2, all groups showed noticeably increased inflammatory cell infiltration. On day 14, the control group exhibited wide ulcerations containing inflammatory cells, a sign of mild inflammation (Figure 3(f)). On day 21, inflammatory cells were observed with little quantity in PYE groups (Figures 3(n) and 3(o)).

Collagen deposition was observed on day 2. On day 14, abundant masses of newly formed collagen had formed on the dermis (Figures 4(f)–4(j)). On day 21, collagen was observed in the dermis in the PYE groups (Figures 4(n) and 4(o)), while collagen fibers filled the dermis in the control group (Figure 4(k)). Collagen content in the PYE groups had significantly increased during the early phases of healing. Day 14 observations suggest that collagen regeneration occurred more efficiently in the PYE groups than in the Vaseline group (Figures 4(h)–4(j)).

### 3.3. Quantification of Interleukin-10 (IL-10)

The wound healing process involves a complex series of overlapping phases that include inflammation, heightened proliferation, and tissue reconstruction [10, 11]. These phases rely on precisely orchestrated interactions between various cells [12]. The inflammatory phase plays an adjunctive role in wound repair by supplying growth factors, cytokines, and chemokines that organize tissue infiltration of peripheral immune cells, such as neutrophils and macrophages, which are required for wound repair. The proliferative phase involves angiogenesis, collagen deposition, connective tissue formation, and wound contraction. Complications that occur during the inflammatory and proliferative phases may disrupt healing after burn injuries, possibly leading to infections and increased morbidity [13].

**Figure 5:**
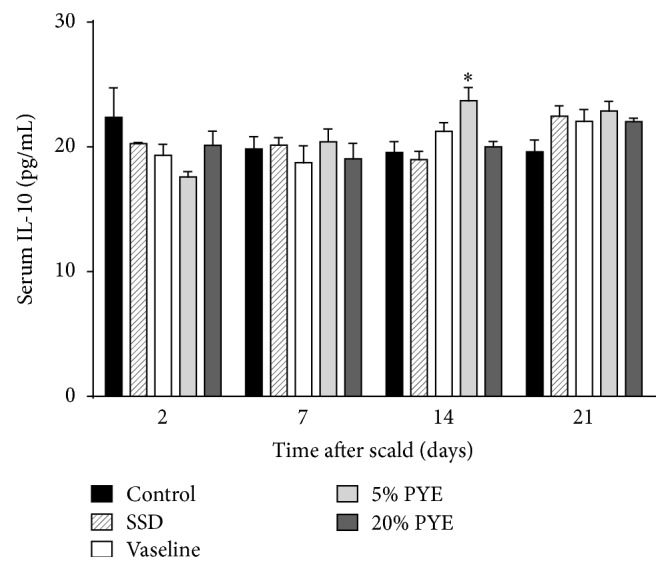
Interleukin-10 (IL-10) levels in cutaneous scalded rat serum. SSD, silver sulfadiazine; PYE,* Prunus yedoensis *Matsumura bark methanol extract. Values were expressed as the mean ± standard error of the mean (*n* = 4–6). ^*∗*^*P* < 0.05 versus control.

Serum samples were collected on days 2, 7, 14, and 21 to determine the impact of the scald wound on the anti-inflammatory response. After 7 days, the IL-10 levels were increased in PYE groups. On day 14, IL-10 level was significantly increased in the PYE groups compared to control group (Figure 5). On day 21, IL-10 levels in all treatment groups were higher than in the control group. IL-10 possesses potent anti-inflammatory properties, inhibiting proinflammatory cytokine production by activated macrophages [14]. In the present study, IL-10 levels were significantly increased on day 14 in the 5% PYE-treated group. This result suggests that PYE could cease or shorten the inflammatory phase at the wound site.

### 3.4. Quantification of Transforming TGF-*β*1

**Figure 6:**
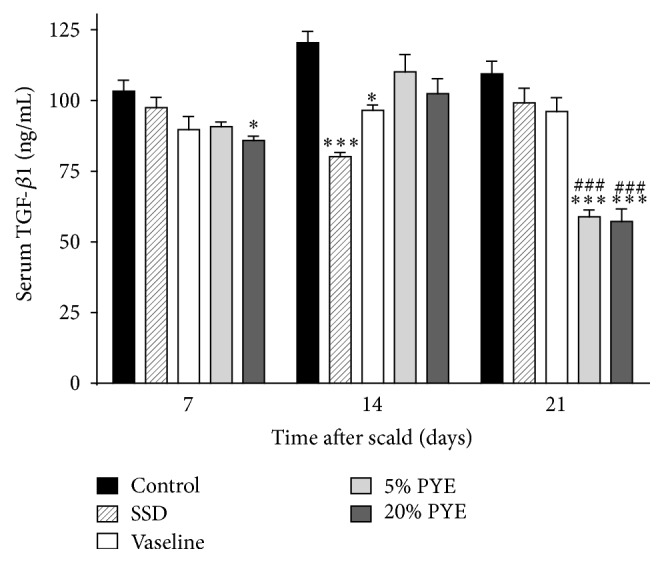
Transforming growth factor beta 1 (TGF-*β*1) levels in cutaneous scalded rat serum. SSD, silver sulfadiazine; PYE,* Prunus yedoensis *Matsumura bark methanol extract. Values were expressed as mean ± standard error of the mean (*n* = 4-5). ^*∗*^*P* < 0.05 and ^*∗∗∗*^*P* < 0.05 versus control. ^###^*P* < 0.001 versus Vaseline.

An increase in TGF-*β*1 was observed between day 7 and day 14 in both of the 5% and 20% PYE groups (90.7 ± 1.7 ng/mL % to 110.1 ± 6.1 ng/mL and 85.8 ± 1.5 ng/mL to 102.4 ± 5.4 ng/mL, resp.), followed by a significant decrease in the levels (to 58.9 ± 2.5 ng/mL and 57.2 ± 4.5 ng/mL, resp.) on day 21 (Figure 6). TGF-*β*1, an important transforming growth factor, is involved in the whole wound healing processes including inflammation, angiogenesis, collagen synthesis, fibroblast proliferation, and remodeling of the new extracellular matrix [15, 16]. It is a proponent of dermal fibrosis and a promoting factor in wound healing processes, and it can improve the rate of healing and wound strength [17, 18]. However, an excess of TGF-*β*1 leads to hypertrophic scarring and keloid formation [17]. Thus, TGF-*β*1 should be increased in the early and interim stages of wound healing process and should be decreased in later stages of the wound healing process. In the present study, TGF-*β*1 levels in both of the 5% and 20% PYE-treated groups increased on day 14 compared to day 7 and then significantly decreased on day 21 in comparison to the other groups. These results, general evaluations (Figures 1 and 2), and histopathological observations (Figures 3 and 4) suggested that PYE could promote wound healing and almost heal scald wounds by day 21 via regulation of TGF-*β*1.

### 3.5. Quantification of VEGF

**Figure 7:**
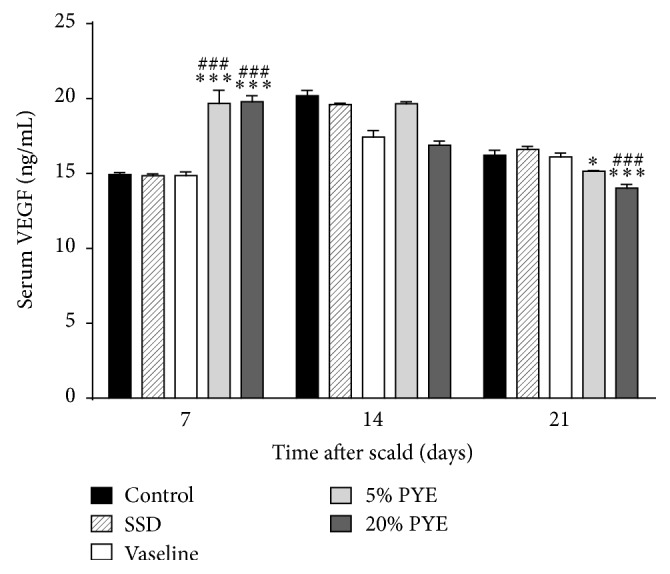
Vascular endothelial growth factor (VEGF) levels in cutaneous scalded rat serum. SSD, silver sulfadiazine; PYE,* Prunus yedoensis *Matsumura bark methanol extract. Values were expressed as mean ± standard error of the mean (*n* = 4–6). ^*∗*^*P* < 0.05 and ^*∗∗∗*^*P* < 0.05 versus control. ^###^*P* < 0.001 versus Vaseline.

Angiogenesis is one of the essential elements of the wound healing process, and VEGF is the most important proangiogenic mediator [19]. VEGF contributes to vascular permeability, affects the interactions between endothelial cells and circulating inflammatory cells, increases the number of dermal mast cells, and plays a role in recruiting macrophages to damaged skin at early stages of healing [19]. In addition, VEGF contributes to wound closure and epidermal repair in the proliferative phase of wound healing. In the final stages of the wound healing process, VEGF promotes scar tissue formation by multiple mechanisms [19]. Thus, VEGF levels should be reduced to reduce scarring. In the present study, on day 7, VEGF levels were significantly higher in both the 5% PYE and 20% PYE groups than in the other groups. As the experiment progressed to day 14 and day 21, the VEGF levels in PYE groups continually dropped. This was a distinctive pattern; other groups showed relatively low VEGF levels until day 7, which increased on day 14 but decreased again on day 21. On day 21, the VEGF levels in PYE groups were significantly lower than in the control group, and the VEGF level in 20% PYE group was significantly lower than in the Vaseline group (Figure 7). These results suggest that PYE could heal scald wounds faster and result in less scarring compared to other treatments, by regulating VEGF in the whole wound healing process.

The TGF-*β*1 and VEGF results from our study suggest that PYE assists and enables unmitigated completion of angiogenesis, reepithelialization, and connective tissue regeneration processes. These findings were further supported by histopathological observations and general evaluation.

Vaseline is a well-known ointment base for burns, wounds, lesions, and other skin conditions [20]. SSD 1% cream is the most widespread topical treatment used in burn injuries due to its antimicrobial efficacy [21]. However, it also has some potential side effects that can lead to delays in the wound healing process and induce serious cytotoxic activity in host cells [22–24]. In the present study, PYE showed better-wound healing effects than Vaseline and SSD in scald wounds.

## 4. Conclusions

PYE showed faster and more effective wound healing activities than SSD and Vaseline in the skin of experimentally scalded rats. Histopathological evaluation results showed better reepithelialization, vascularization, granulation tissue formation, and collagen deposition in the PYE groups than the other groups. PYE application to scald wounds resulted in less scarring than the other treatments. These effects were due to the appropriate regulation of IL-10, TGF-*β*1, and VEGF.
